# Conduction system pacing as a rescue strategy for unsuccessful cardiac resynchronisation therapy implantation

**DOI:** 10.3389/fphys.2026.1929170

**Published:** 2026-09-16

**Authors:** Kaung Myat Thu, Ibrahim Antoun, Kyi Zin Thant, Ahmed Abdelrazik, Mahmoud Eldesouky, Hamza Tarig Hamza, Sharon Man, Zakariyya Vali, Riyaz Somani, G. André Ng, Mokhtar Ibrahim

**Affiliations:** 1 Department of Cardiology, University Hospitals of Leicester NHS Trust, Glenfield Hospital, Leicester, United Kingdom; 2 Department of Cardiovascular Sciences, Clinical Science Wing, University of Leicester, Glenfield Hospital, Leicester, United Kingdom; 3 National Institute for Health Research Leicester Research Biomedical Centre, Leicester, United Kingdom; 4 Leicester British Heart Foundation Centre of Research Excellence , Glenfield Hospital, Leicester, United Kingdom

**Keywords:** conduction system pacing, heart failure, HIS bundle pacing, left bundle branch area pacing, unsuccessful cardiac resynchronisation therapy

## Abstract

**Background:**

Failure to achieve effective cardiac resynchronisation therapy (CRT) remains a clinically significant challenge, often driven by anatomical constraints or suboptimal left ventricular lead performance. Conduction system pacing (CSP), incorporating His bundle pacing and left bundle branch area pacing, has emerged as a physiological alternative; however, data focussing specifically on its role as a rescue strategy following unsuccessful CRT implantation, rather than biological non-response, remain limited.

**Objective:**

To evaluate the feasibility, safety, and short- to mid-term clinical and echocardiographic outcomes of CSP in patients with unsuccessful conventional CRT implantation.

**Methods:**

In this single-centre retrospective study, consecutive patients undergoing attempted CSP after unsuccessful CRT between January 2020 and May 2025 were analysed. Unsuccessful CRT was defined as inability to deliver effective biventricular pacing due to anatomical or technical limitations, excluding patients with true non-response despite successful implantation. Procedural success, electrical resynchronisation, changes in left ventricular ejection fraction (LVEF), and clinical outcomes were assessed.

**Results:**

Thirty-two patients (mean age 69 years, mean LVEF 27.8%) were included. CSP was successfully achieved in 91% of cases, predominantly using left bundle branch area pacing. Baseline QRS duration (165 ± 38 ms) significantly narrowed to 122 ± 19.8 ms following CSP. This was accompanied by marked improvement in LVEF (from 27.0 ± 8.6% to 47.7 ± 11.2%, p < 0.001). Procedural complications were infrequent, with one pneumothorax and no device-related infections. Over a mean follow-up of 641 ± 522 days, heart failure hospitalisation occurred in 7%, and three deaths were observed, non-attributable to device-related causes.

**Conclusion:**

CSP is a highly feasible and safe rescue strategy in patients with unsuccessful CRT implantation, achieving substantial electrical resynchronisation and meaningful recovery of left ventricular function. These findings support CSP as a compelling alternative in anatomically or technically challenging CRT cases where conventional resynchronisation delivery is not achievable.

## Introduction

Cardiac resynchronisation therapy (CRT) has become a foundational intervention for patients with symptomatic heart failure and evidence of electrical dyssynchrony, particularly those with reduced left ventricular ejection fraction and prolonged QRS duration. Landmark clinical trials have consistently demonstrated that conventional biventricular pacing improves quality of life, reduces heart failure hospitalisations, and decreases all-cause mortality compared with standard medical therapy alone (Cazeau et al., 2001; Abraham et al., 2002; Bristow et al., 2004). Despite these well-established benefits, a substantial proportion of patients do not achieve meaningful clinical or echocardiographic improvement following conventional treatment, and anatomical or technical limitations may preclude optimal left ventricular lead placement via the coronary sinus. Observational data and systematic reviews estimate that up to one-third of patients receiving biventricular pacing fail to respond adequately to therapy, and that unsuccessful or complicated left ventricular lead implantation occurs in a small but clinically significant subset of patients (Hua et al., 2022).

In patients in whom conventional coronary sinus left ventricular lead implantation is unsuccessful, the historical bailout option for achieving resynchronisation has been surgical epicardial left ventricular lead placement. Although this approach can provide clinical benefit, it is more invasive, typically requires surgical access and general anaesthesia, and has been associated with perioperative complications including hypotension, major bleeding, stroke, acute renal failure and prolonged recovery in an already high-risk heart failure population (Shah et al., 2006; Patwala et al., 2009; Ailawadi et al., 2010).

Conduction system pacing (CSP) has developed as a conceptually attractive alternative to bypass some of the intrinsic limitations of conventional approaches (Wouters et al., 2021). His bundle pacing was the first physiological pacing technique described (Deshmukh et al., 2000), enabling direct activation of the conduction system but limited by technical complexity, variable success rates, and challenges with pacing thresholds. This led to the development of left bundle branch area pacing (LBBAP), which targets a more distal segment of the conduction system and appears to provide physiological activation with higher procedural success rates and more stable electrical parameters (Huang et al., 2017). **Figure 1**. A growing body of observational evidence suggests that LBBAP may achieve electrical and mechanical resynchronisation that is at least comparable to, and in some cases superior to, CRT, with narrower paced QRS complexes, greater improvements in left ventricular function, and favourable clinical responses in heart failure symptoms (Sharma et al., 2018). Although large-scale randomised controlled trials are not yet available, these data support the physiological premise that restoring near-native activation patterns via conduction system pacing may confer meaningful benefits beyond those achievable through conventional strategies. Current clinical consensus and expert opinion increasingly recognise CSP as a viable alternative or adjunct to biventricular pacing, particularly in patients with anatomical constraints, non-response to conventional therapy, or non-left bundle branch block conduction delay, while emphasising the need for further evidence from prospective comparative studies (Domenichini et al., 2023). A recent meta-analysis of CSP in patients with heart failure and non-left bundle branch block morphology demonstrated that CSP is associated with significant improvement in left ventricular systolic function, reverse ventricular remodelling, amelioration of heart failure symptoms, and restoration of electrical synchrony (Thu et al., 2025).

**Figure 1:**
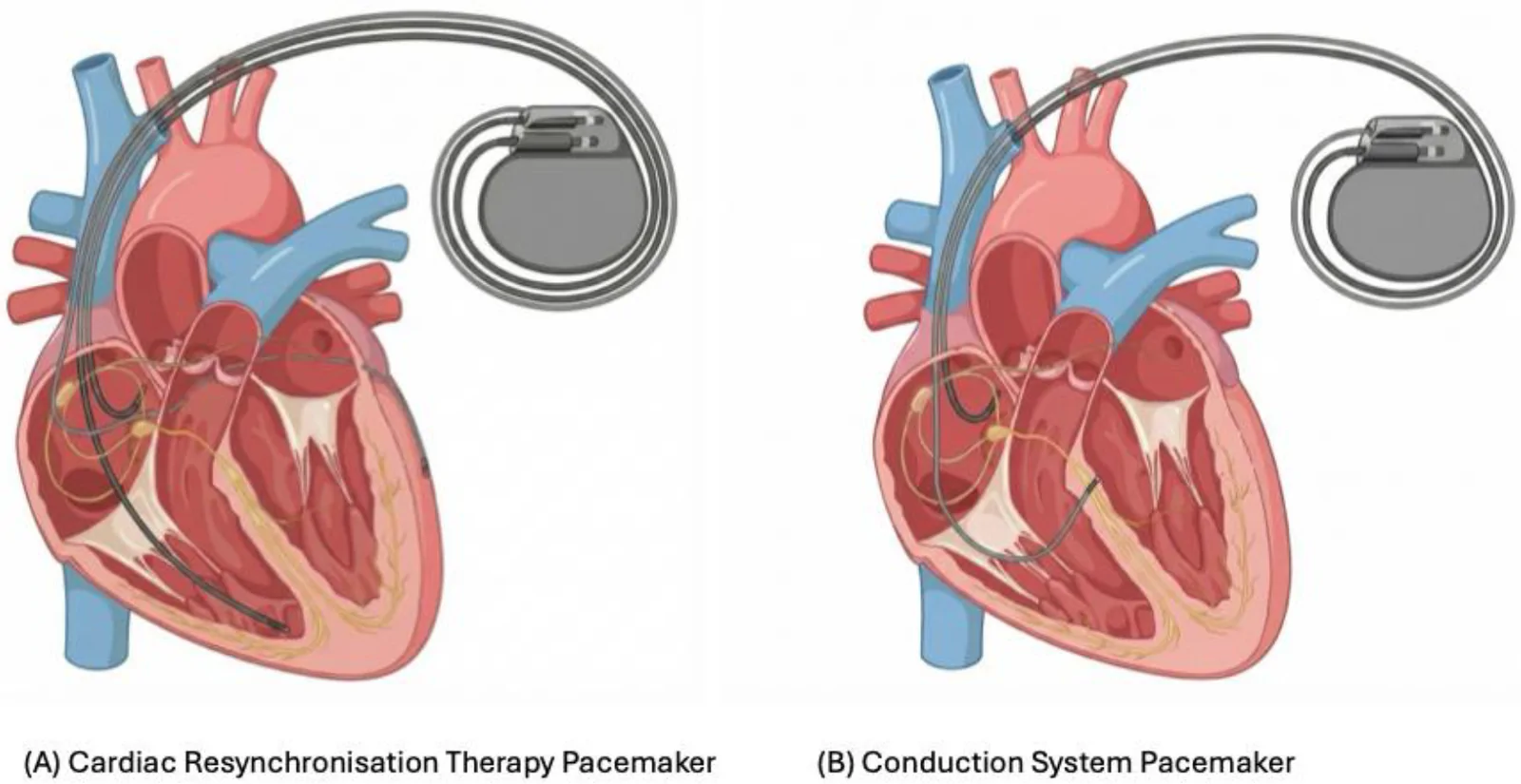
Illustration of **(A)** cardiac resynchronisation therapy (CRT) pacemaker. **(B)** conduction system pacemaker (CSP).

While accumulating evidence suggests that CSP can deliver favourable electrical and echocardiographic outcomes, most available data derive from heterogeneous cohorts or mixed indications, and there remains limited focussed evidence addressing its role specifically as a bailout strategy following unsuccessful CRT. Furthermore, real-world data describing procedural feasibility, safety, and functional recovery in patients with prior failed resynchronisation attempts are scarce. This study was therefore undertaken to address this knowledge gap by evaluating the outcomes of CSP in a carefully characterised cohort of patients with unsuccessful conventional CRT managed at a high-volume tertiary centre.

## Methods

### Study design

This was a retrospective observational study conducted at a single tertiary referral centre. Clinical, procedural, electrocardiographic, and echocardiographic data were collected from institutional databases and electronic medical records. All eligible patients who underwent attempted conduction system pacing after unsuccessful conventional CRT between January 2020 and May 2025 were included. The study was designed to reflect real-world clinical practice, with procedural strategy and pacing modality determined by operator discretion and anatomical considerations. The study protocol was approved in accordance with local institutional governance requirements.

### Study population

The present study deliberately focussed on patients with failed CRT due to technical or anatomical limitations of lead implantation, rather than patients with true non-response despite technically successful CRT delivery. Individuals who underwent CRT implantation with satisfactory lead position and electrical parameters but failed to demonstrate clinical or echocardiographic response were therefore not included. This distinction was made to reduce heterogeneity, avoid potential misclassification, and strengthen the internal validity of the analysis by isolating a cohort in whom CRT failure was primarily procedural rather than biological. Therefore, the study population comprised consecutive patients with heart failure and reduced left ventricular ejection fraction who had previously undergone unsuccessful conventional CRT.

During the predefined study period, patients with unsuccessful CRT implantation subsequently underwent attempted conduction system pacing, using either His bundle pacing or left bundle branch area pacing. Baseline demographic, clinical, electrocardiographic, and echocardiographic characteristics were recorded at the time of implantation of the conduction system pacemaker, with follow-up data obtained from routine clinical assessments and device evaluations.

### Definition of unsuccessful cardiac resynchronisation therapy

Unsuccessful CRT is generally defined as the inability to deliver effective, durable biventricular pacing despite an appropriate clinical indication. This includes failure to implant a left ventricular lead via the coronary sinus because of unfavourable venous anatomy, inability to cannulate the coronary sinus, or procedural complications such as coronary sinus dissection. It also encompasses cases in which a left ventricular lead is implanted but where effective resynchronisation cannot be achieved due to unacceptably high pacing thresholds, failure to achieve consistent myocardial capture, unavoidable phrenic nerve stimulation, lead instability or displacement, or device-related infection requiring lead extraction. In addition, some definitions include patients with persistent electrical dyssynchrony or lack of echocardiographic or clinical response despite technically successful implantation.

Five experienced CRT implanters performed procedures during the study period. As this was intended to represent real world practice, no standardised institutional protocol existed regarding the duration of CRT attempts or the sequence of techniques used for coronary sinus cannulation. The choice of delivery sheath, guidewire, electrophysiology catheter, venography technique, alternative venous access, or lead delivery strategy was determined by the individual operator.

Following unsuccessful CRT implantation, subsequent management was individualised. Cases recognised after the procedure were discussed at the multidisciplinary device meeting, where decisions regarding CSP, conventional pacing, or conservative management were based on patient comorbidities, clinical indication, and anticipated pacing requirements. When CRT implantation failed during the index procedure, immediate conversion to CSP was at the discretion of the implanting operator. Data regarding patients with unsuccessful CRT who were managed without CSP were not systematically collected.

Our institution is a high-volume tertiary referral centre with an overall conventional CRT implantation success rate consistently exceeding 90%, comparable to contemporary published series. The present study therefore represents a highly selected cohort in whom conventional CRT implantation was unsuccessful despite standard procedural attempts.

### Definition of successful conduction system pacing

Successful conduction system pacing is defined by stable lead implantation with reliable capture of the native cardiac conduction system at acceptable pacing thresholds, resulting in physiological ventricular activation.

For HBP, success is defined as selective or non-selective His bundle capture, evidenced by paced QRS morphology identical or nearly identical to the intrinsic QRS, or by correction of bundle branch block with narrowing of the QRS duration. Additional criteria include stable sensing, acceptable capture thresholds, and absence of lead-related complications. These definitions are well described in consensus documents and clinical studies by Vijayaraman et al. (2023).

For LBBAP, successful implantation is defined by lead deployment deep within the interventricular septum with capture of the left bundle branch or its proximal fascicles. Electrophysiological criteria include a paced right bundle branch block pattern in lead V1, a short and stable left ventricular activation time, demonstration of left bundle branch potential or a transition from non-selective to selective capture, where feasible, and significant QRS narrowing compared with baseline. Stable pacing thresholds and adequate sensing are also required. These criteria are described in foundational studies of LBBAP (Huang et al., 2017). All CSP procedures were performed using a lumenless Medtronic SelectSecure 3830 pacing lead. No stylet driven CSP leads were used during the study period. Conduction system pacing was performed by three experienced CSP operators. Deep septal myocardial pacing without evidence of conduction system capture was not classified as successful CSP.

Successful CSP required stable conduction system capture with acceptable pacing thresholds. In our practice, thresholds exceeding approximately 2.5 V at 0.4 ms for LBBAP or 2.5 to 3.0 V at 1.0 ms for HBP were considered unsuitable for long term implantation because of concerns regarding battery longevity and chronic lead performance. Device programming was individualised according to the implanted system, underlying rhythm and clinical indication, with CSP programmed to provide ventricular resynchronisation. In patients receiving CRT-D systems, conventional defibrillation capability was maintained.

### Outcomes

The primary objective of this study was to assess the feasibility and procedural success of CSP in patients who had failed conventional CRT. Secondary objectives were to evaluate the impact of CSP on electrical resynchronisation, as assessed by changes in QRS duration, and on left ventricular systolic function, as assessed by changes in left ventricular ejection fraction. Additional objectives included assessing procedural complications and subsequent hospitalisation events during follow-up to provide a comprehensive evaluation of safety and early clinical outcomes in this challenging patient population.

### Statistical analysis

Categorical variables were expressed as frequency and percentage. The mean ± standard error of the mean was adopted to describe continuous parametric data. Pearson’s χ 2 or Fisher’s exact test was used to compare categorical variables between groups. Paired Students’ t-tests and Kruskal-Wallis tests were used to compare continuous variables between groups, depending on the distributional normality. A 2-sided p-value <0.05 was considered statistically significant. Statistical analysis was performed using GraphPad Prism V10.3 for Mac (San Diego, California, USA).

## Results

A total of 32 patients with failed or unsuccessful CRT implants were included in the analysis. The cohort was predominantly male, with 21 men, and had a mean age of 69 years, reflecting an elderly population with advanced structural heart disease. Comorbid conditions were common, including ischaemic heart disease in 37%, atrial fibrillation in 41%, diabetes mellitus in 22%, and hypertension in 25%. At baseline, patients were moderately symptomatic, with a mean New York Heart Association functional class of 2.6, and exhibited severe left ventricular systolic dysfunction, with a mean left ventricular ejection fraction of 27.8%. **Table 1**.

**Table 1:** Baseline characteristics.

Demographic profile	N = 32
Age (y)	69±14
Sex	21 (male)
Diabetes	7 (22%)
Hypertension	8 (25%)
IHD	12 (37%)
AF	13 (41%)
Mean baseline functional NYHA class	2.6
LVEF (%)	27±8.6
Electrocardiographic parameters	
Baseline QRS duration (ms)	165±38
LBBB morphology	20 (62%)
RBBB morphology	5 (16%)
Narrow QRS	5 (16%)
RV paced	2 (6%)
Unsuccessful CRT reason	
Difficult CS anatomy, CS cannulation	15 (47%)
Unsatisfactory LV lead parameters (high threshold, failure to capture, phrenic nerve stimulation)	11 (35%)
CIED infection requiring extraction	3 (9%)
CS dissection	1 (3%)
LV lead displacement	1 (3%)
Left sided SVC	1 (3%)

Values are presented as meanSD or n (%).

AF, atrial fibrillation; CIED, cardiac implantable electronic device; CRT, cardiac resynchronisation therapy; CS, coronary sinus; EF, ejection fraction; IHD, Ischemic heart disease; LBBB, left bundle branch block; LV, left ventricle; NYHA, New York Heart Association; RBBB, right bundle branch block; RV, right ventricle.

Electrocardiographically, patients demonstrated significant electrical dyssynchrony, with a markedly prolonged baseline QRS duration of 165 ms. LBBB morphology was the predominant pattern, present in 62% of cases, while RBBB and narrow QRS complexes were observed in 16% each. A minority of patients (6%) were right-ventricular-paced at baseline.

Lead performance parameters were favourable at implantation, with a mean R wave sensing amplitude of 7.40 ± 4.35 mV and stable values at follow up (7.11 ± 5.03 mV). Capture thresholds were low at implantation (0.88 ± 0.39 V) and remained stable at follow up (0.78 ± 0.33 V), with no evidence of clinically significant threshold rise over time. Lead impedance was within acceptable ranges at implantation (701 ± 155 Ω) and demonstrated a reduction at follow up (478 ± 123 Ω), remaining within physiological limits and without evidence of lead dysfunction.

Among patients undergoing successful LBBAP, all demonstrated evidence of left bundle branch capture and were classified as LBBP, with no cases of isolated LVSP. Nine patients initially underwent attempted HBP but were crossed over to LBBAP during the same procedure because of an unacceptably high HBP threshold. Among successful LBBAP cases with available electrical measurements, the mean LVAT was 85.58 ms and the mean RWPT was 39.5 ms. In patients with baseline LBBB morphology, mean LVAT was 82.57 ± 5.38 ms and mean RWPT was 41.2 ± 9.55 ms. In patients with non LBBB morphology, mean LVAT was 89.8 ± 11.50 ms and mean RWPT was 36.67 ± 7.02 ms.

Procedural metrics demonstrated a mean fluoroscopy time of 17.3 ± 15.5 minutes and a median radiation dose of 631 mGy·cm², with variability reflecting case complexity.

The reasons for the failure of conventional CRT were heterogeneous. The most frequent cause was challenging coronary sinus anatomy or failure of coronary sinus cannulation, accounting for 47% of cases. Suboptimal left ventricular lead performance, including high capture thresholds, inability to capture, or phrenic nerve stimulation, was observed in 34%. Less common causes included cardiac implantable electronic device infection requiring extraction, coronary sinus dissection, left ventricular lead displacement, and the presence of a left-sided superior vena cava. **Figure 2**. Device related infection was not considered a technical failure of CRT implantation. Rather, these patients required complete system extraction and subsequent reimplantation, during which CSP was selected as the alternative physiological pacing strategy instead of repeat coronary sinus lead implantation.

**Figure 2:**
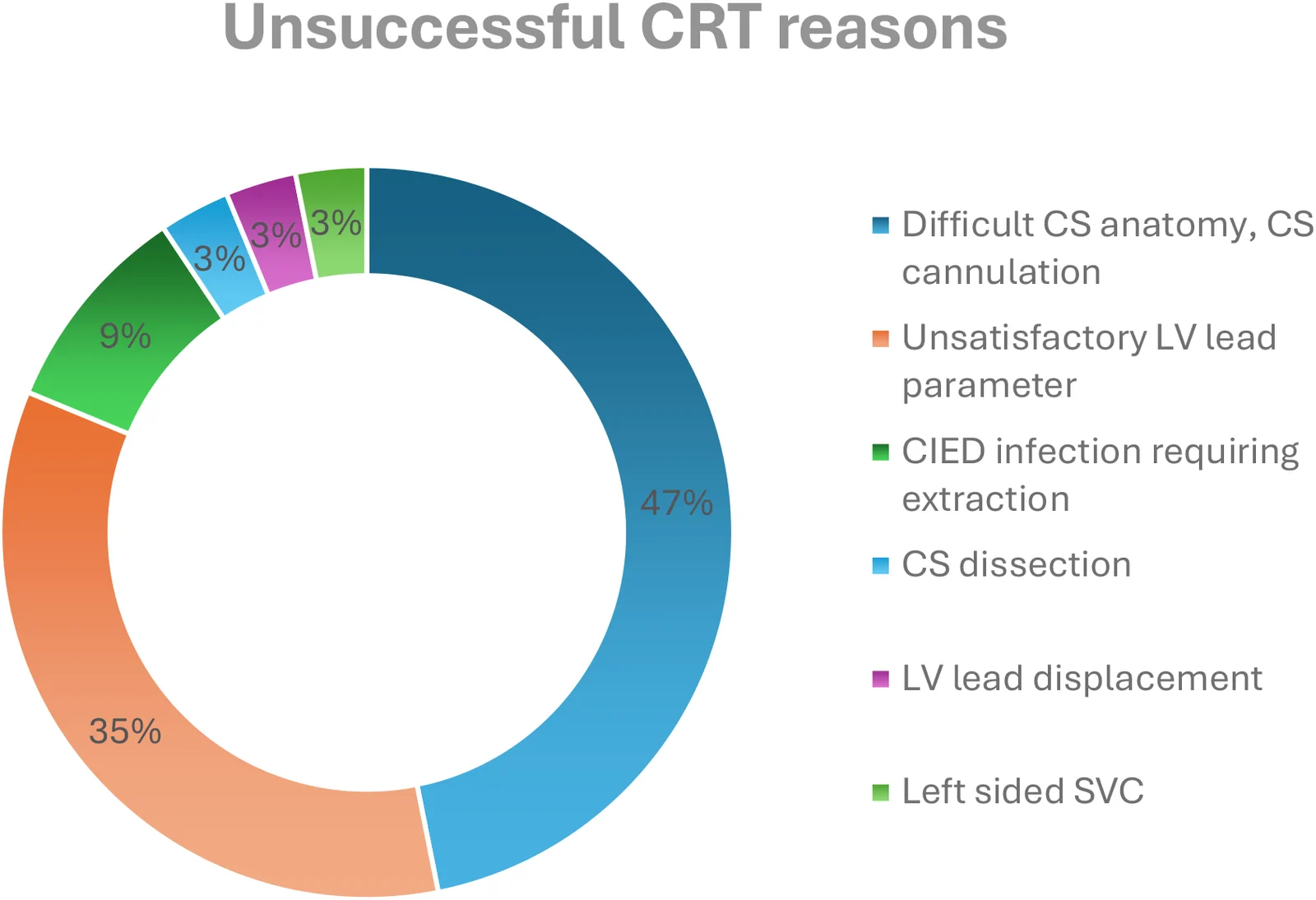
Unsuccessful CRT implant reasons in percentage. CIED, Cardiac Implantable Electronic Device; CRT, Cardiac Resynchronisation Therapy; CS, coronary sinus; LV, left ventricle; SVC, Superior Vena Cava.

CSP was successfully achieved in 29 of the 32 patients, corresponding to an overall procedural success rate of 91%. Three cases of unsuccessful CSP implantation were attributable to suboptimal pacing parameters. These included one case of an unacceptably high capture threshold related to an underlying myocardial scar, and two cases in patients with adult congenital heart disease who had previously undergone ventricular septal defect repair procedures. Among successful cases, LBBAP was the most frequently employed strategy (72%), whereas HBP was performed in 28%. **Table 2**.

**Table 2:** Procedural outcomes.

Successful CSP implementation	29 (91%)	*P value*
Type conduction system pacing		
LBBAP	21 (72%)	
HBP	8 (28%)	
Pre-CSP LVEF (%)	27±8.6	*p*<0.0001
Post-CSP LVEF (%)	47.7±11.2	
Pre-CSP QRS duration	165±38 ms	*p*<0.0001
Post-CSP QRS duration	122±19.8 ms	
R wave sensing at baseline	7.40±4.35 mV	
R wave sensing at follow up	7.11±5.03 mV	
Threshold at baseline	0.88±0.39 V	
Threshold at follow up	0.78±0.33 V	
Lead impedance at baseline	701±155 Ω	
Lead impedance at follow up	478±123 Ω	
RWPT (mean)	39.5 ms	
LVAT (mean)	85.58 ms	
Fluoroscopy time (mean)	17.3±15.5 min	
Radiation dose (median)	631 mGy·cm²	
Complications		
Pneumothorax	1 (3%)	
Pericardial effusion	0 (0%)	
Bleeding (minor, major)	0 (0%)	
Device infection	0 (0%)	
Hospitalisation	8 (28%)	
Heart failure Hospitalisation	2 (7%)	
Non-cardiac chest pain	2 (7%)	
Fall	2 (7%)	
VT ablation	1 (3%)	
Gastro-enteritis	1 (3%)	

Values are presented as n (%).

CSP, conduction system pacemaker; HBP, His bundle pacing; LBBAP, left bundle branch area pacing; LVAT, left ventricular activation time; LVEF, left ventricular ejection fraction; RWPT, R-wave peak time; VT, ventricular tachycardia.

At baseline, the pre-implantation electrocardiogram demonstrated left bundle branch block in 20 patients (62%), right bundle branch block in 3 patients (16%), a narrow QRS complex in 4 patients (16%) with underlying severe left ventricular systolic dysfunction, and a ventricular paced rhythm in 3 patients (6%). In the narrow QRS cohort, the indication for cardiac resynchronisation therapy was primarily driven by the presence of severe left ventricular systolic dysfunction in the context of atrial fibrillation, where a pace and ablate strategy was pursued to mitigate the risk of further ventricular deterioration that may arise following conventional dual chamber pacing. In addition, a proportion of patients developed a high burden of right ventricular pacing, either as a consequence of device therapy or following atrioventricular node ablation, which further supported the need for resynchronisation. Other contributing indications included cases of cardiac implantable electronic device infection requiring system revision, as well as suboptimal pacing parameters that precluded effective conventional pacing and necessitated an alternative physiological pacing approach.

Pre-CSP mean QRS duration was 165 ± 38 ms and mean left ventricular ejection fraction was 27 ± 8.6%. Following CSP, there was significant electrical resynchronisation, with QRS duration narrowing to 122 ± 19.8 ms and a marked improvement in left ventricular systolic function, with LVEF increasing to 47.7 ± 11.2%; both changes were highly statistically significant. **Figure 3**.

**Figure 3:**
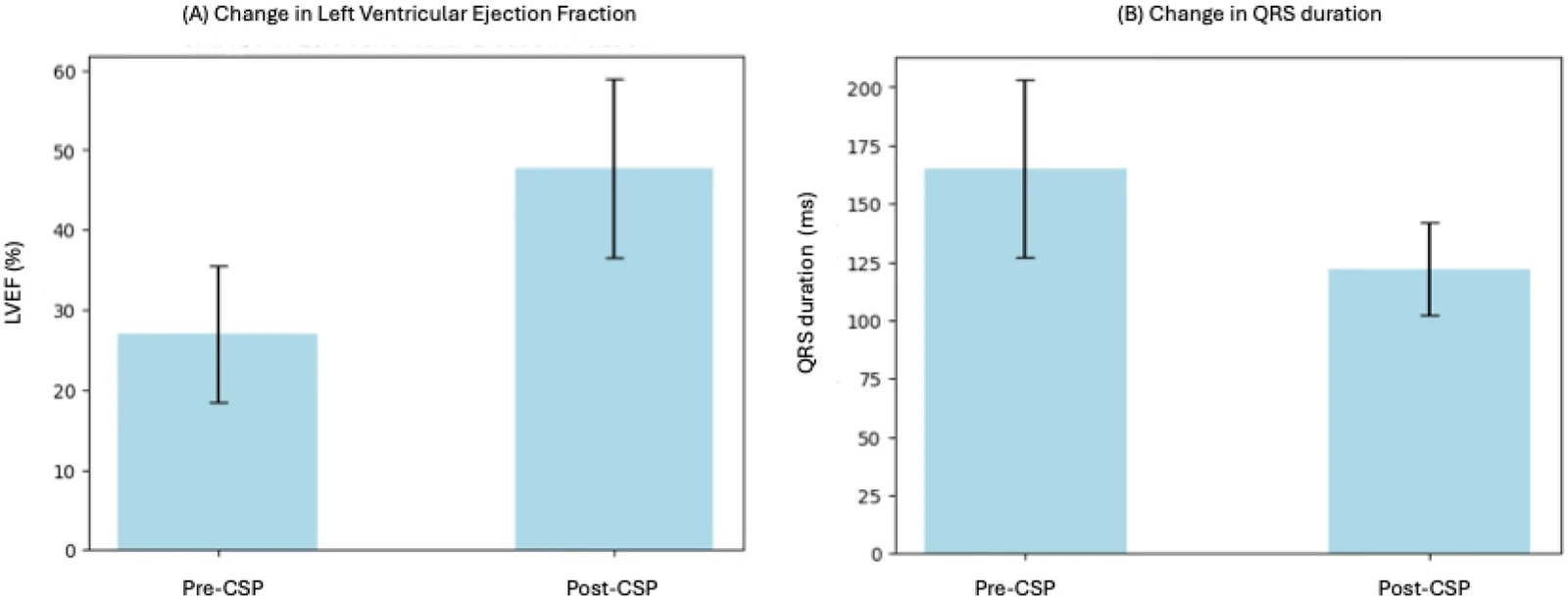
Electrical and echocardiographic remodeling following conduction system pacing. **(A)** Mean left ventricular ejection fraction pre-CSP 27±8.6%and post-CSP 47.7±11.2% (p<0.0001) implantation. **(B)** Mean QRS duration pre-CSP 165±38 ms and post-CSP 122±19.8 ms (p<0.0001) implantation. Error bars represent standard deviation. Comparisons were performed using paired statistical testing.

Procedural complications were infrequent. One patient experienced a pneumothorax, while no cases of pericardial effusion, clinically significant bleeding, or device-related infection were observed. During follow-up, 28% of patients required hospitalisation. Heart failure-related admissions occurred in 7%, while other admissions were attributable to non-cardiac chest pain, falls, ventricular tachycardia ablation, and gastroenteritis.

During follow-up, three patients died. Two deaths were attributed to progressive heart failure and one to a non-cardiac cause. Among those who died from heart failure, one patient had advanced ischaemic cardiomyopathy with coexistent atrial fibrillation, while the other had severe left ventricular systolic dysfunction of non-ischaemic aetiology. In both cases, clinical deterioration occurred despite optimisation of guideline-directed medical therapy, with no evidence of device-related complications or dysfunction. Both patients who died from heart failure had previously achieved successful CSP implantation with stable pacing parameters and maintained conduction system capture throughout follow up. The cohort was followed for a mean of 641 ± 522 days, indicating substantial variability in follow-up duration and capturing both short- to mid-term clinical outcomes.

## Discussion

Historically, when transvenous coronary sinus lead implantation was not feasible, surgical epicardial left ventricular lead placement represented the principal rescue strategy for CRT delivery. While surgical epicardial leads can achieve comparable haemodynamic and clinical benefit in selected patients, this approach is inherently more invasive and may require general anaesthesia, thoracotomy or thoracoscopic access, perioperative monitoring, and longer postoperative recovery, all of which may be undesirable in patients with advanced heart failure and multiple comorbidities (Navia et al., 2005; Patwala et al., 2009; Ailawadi et al., 2010; Marini et al., 2020). In contrast, conduction system pacing, particularly left bundle branch area pacing, offers a transvenous and less invasive alternative that is now recognised in contemporary pacing guidance as a rescue strategy when coronary sinus lead implantation is unsuccessful (Glikson et al., 2021; Glikson et al., 2025).

The concept of physiological pacing, achieved by engaging the intrinsic conduction system, has emerged in response to the limitations of conventional techniques. CSP, encompassing HBP and LBBAP, aims to recruit the native His-Purkinje network to achieve near-physiological ventricular activation and more complete electrical synchrony. To the best of our knowledge, the present study represents an important systematic evaluation of conduction system pacing as a therapeutic strategy in patients with failed cardiac resynchronisation therapy, thereby extending the application of physiological pacing to a population with limited alternative options and setting the framework for the observations discussed below.

Importantly, only physiological conduction system pacing techniques, including His bundle pacing and left bundle branch area pacing meeting accepted electrophysiological criteria, were included in this study. Deep septal myocardial pacing without evidence of conduction system capture was not considered successful CSP. The haemodynamic response to CSP is also likely to depend upon the underlying myocardial substrate, with differences expected between ischaemic, dilated, pacing induced and other cardiomyopathies. These factors should be considered when interpreting the observed clinical response.

Early observational studies and meta-analyses suggest that CSP can produce greater QRS narrowing, superior reverse remodelling, and improvements in left ventricular ejection fraction when compared with conventional biventricular pacing. Notably, a recent systematic review and network meta-analysis found that CSP was associated with greater improvements in QRS duration and echocardiographic response, and suggested potential reductions in mortality and in the risk of heart failure hospitalisation (Tavolinejad et al., 2023). Furthermore, in patients with non-left bundle branch block morphology, CSP has been associated with more frequent echocardiographic response and improved outcomes when compared with biventricular pacing in prospective registries, reinforcing the physiological rationale for this approach across diverse conduction substrates (Thu et al., 2025).

This study demonstrates that CSP is a highly effective and safe rescue strategy in patients with unsuccessful conventional CRT, leading to substantial improvements in both ventricular electrical synchrony and systolic function. The observed procedural success rate of over 90 per cent is particularly notable given the technical challenges and anatomical constraints that precluded effective biventricular pacing in this cohort. The baseline characteristics highlight a population with advanced disease and a high burden of comorbidity, typical of patients referred for CRT. The predominance of left bundle branch block and markedly prolonged baseline QRS duration underscores significant electrical dyssynchrony, a key therapeutic target in resynchronisation therapy.

Despite its widespread adoption, conventional CRT is constrained by its reliance on coronary venous anatomy to deliver left ventricular pacing. Anatomical variability, the absence of suitable lateral or posterolateral venous branches, and technical challenges in cannulating the coronary sinus may render left ventricular lead placement suboptimal or unsuccessful. In addition, even when lead implantation is technically achieved, factors such as high pacing thresholds, phrenic nerve stimulation, and chronic lead instability can limit effective resynchronisation (Pires et al., 2014). Importantly, in this study, nearly half of CRT failures were attributable to coronary sinus anatomy, underscoring a fundamental limitation of conventional CRT that is independent of patient selection and reflects procedural and anatomical constraints.

CSP directly addresses these limitations by engaging the intrinsic His-Purkinje system, thereby obviating the need for coronary sinus lead placement. The substantial reduction in QRS duration observed in this study provides mechanistic evidence of restored physiological activation. This electrical resynchronisation resulted in meaningful reverse remodelling, as evidenced by a marked improvement in left ventricular ejection fraction (Ezzeddine et al., 2023). The magnitude of LVEF recovery observed here compares favourably with, and in some cases exceeds, that reported in conventional CRT responder populations, despite this cohort having previously failed CRT.

The predominance of left bundle branch area pacing reflects contemporary practice and growing operator familiarity with this technique. LBBAP provides more stable lead parameters and lower capture thresholds than HBP, which may explain its preferential use in this complex population (Vijayaraman et al., 2021). Nevertheless, the successful use of HBP in over a quarter of cases highlights its continued relevance, particularly in selected anatomical or conduction scenarios.

The low complication rate observed supports the procedural safety of CSP, even in patients with prior device interventions and complex venous anatomy. The limited number of heart failure hospitalisations during follow-up further suggests that the physiological benefits of CSP are sustained beyond the acute procedural period. However, longer-term follow-up is required to confirm durability.

Taken together, these findings support CSP as a robust and clinically meaningful alternative for patients with failed CRT. By overcoming the anatomical and technical limitations of coronary sinus-based pacing, CSP offers a path towards true physiological resynchronisation. In an era where non-response to CRT remains a significant clinical challenge, this strategy has the potential to redefine rescue therapy and may warrant earlier consideration in selected patients. Future prospective and randomised studies are required to confirm these findings and to refine patient selection, pacing strategy, and long-term outcomes.

The findings of this study should be interpreted within the context of existing evidence on conduction system pacing as an alternative approach to cardiac resynchronisation therapy. Conventional CRT remains the established standard for patients with symptomatic heart failure, reduced left ventricular ejection fraction, and prolonged QRS duration, with landmark trials demonstrating significant reductions in mortality and heart failure events. Nevertheless, up to one-third of patients are considered non-responders to conventional therapy, due in part to limitations in electrical synchronisation and coronary venous anatomy, which may preclude optimal left ventricular lead placement. Systematic reviews and meta-analyses confirm that cardiac resynchronising with CSP, incorporating both HBP and LBBAP, can achieve greater electrical synchrony and meaningful improvements in left ventricular function compared with biventricular pacing, despite the heterogeneous methodologies of available studies (Tavolinejad et al., 2023). A recent meta-analysis demonstrated that CSP was associated with a substantial reduction in QRS duration, an improvement in ejection fraction, and a decrease in symptomatic class compared with biventricular pacing, noting that further randomised trials are needed to establish long-term efficacy and safety (Gin et al., 2023). Additional observational evidence suggests that clinical outcomes, including mortality and heart failure hospitalisation, may be lower with CSP compared to biventricular pacing, particularly in patients with left bundle branch block morphology, supporting the concept that a more physiological activation sequence can translate into superior clinical benefit. In an observational cohort of 477 patients, paced QRS duration was significantly narrower in those receiving conduction system pacing than in those receiving conventional biventricular pacing, and left ventricular ejection fraction and the composite outcome of death or heart failure hospitalisation were more favourable with conduction system pacing (Vijayaraman et al., 2022).

Device strategies incorporating CSP continue to evolve, particularly in patients requiring defibrillator therapy. More recently, integrated defibrillator and LBBAP lead technologies have emerged as a potential approach to combine physiological ventricular activation and defibrillation within a simplified lead configuration. Further evidence regarding the long-term safety, lead performance and clinical outcomes of these systems will be required before their broader adoption.

The relatively large standard deviation for follow up reflects the inclusion of consecutive patients treated over a five-year period, resulting in varying observation times according to implantation date. Although all available clinical, echocardiographic, electrocardiographic, and device follow up data were included, this variability may have limited the detection of very late complications or changes in long term device performance. Prospective studies with predefined follow up intervals are therefore warranted.

Considering these data, the substantial improvements in QRS duration, left ventricular ejection fraction, and procedural feasibility observed in the present cohort align with the broader evidence base that supports conduction system pacing as an effective alternative for patients in whom conventional cardiac resynchronisation therapy has failed or is not feasible.

### Limitation of the study

Several limitations of this study should be acknowledged. First, the retrospective observational design and single-centre setting introduce inherent risks of selection bias and unmeasured confounding, limiting causal inference and generalisability to other populations or practice settings. Second, the sample size was modest, reflecting the specialised nature of failed CRT referrals, which reduces statistical power and precludes robust subgroup analyses comparing HBP with LBBAP. Third, the study lacked a contemporaneous control group of patients managed with alternative strategies, such as repeat coronary sinus lead attempts or surgical epicardial pacing, thereby limiting comparative effectiveness conclusions. Finally, by design, the cohort excluded patients with true non-response despite technically successful CRT implantation, thereby strengthening internal validity but limiting extrapolation of the findings to the broader population of CRT non-responders. Prospective multicentre studies with larger cohorts and longer follow-up are therefore required to confirm these findings and better define the role of conduction system pacing in the failed CRT population.

An additional limitation is the heterogeneous underlying cardiomyopathy within the study population. As with conventional CRT, response to CSP may vary according to myocardial substrate, including ischaemic, dilated, pacing induced or valvular cardiomyopathy. Furthermore, no contemporaneous comparator cohort was available for propensity matched analysis with successful CRT recipients or conventional right ventricular pacing, limiting comparative effectiveness analyses.

### Future directions

Further research is required to define the optimal role of CSP in heart failure and the cardiac resynchronisation therapy landscape. Prospective randomised trials comparing CSP with conventional biventricular pacing, both as a primary strategy and as a bailout approach, are essential to establish comparative efficacy, durability of benefit, and long-term clinical outcomes, including mortality and heart failure hospitalisation. Studies incorporating standardised definitions of procedural success, electrical resynchronisation, and clinical response will be critical to allow meaningful comparison across pacing modalities.

In addition, future work should focus on refining patient selection criteria to identify subgroups most likely to benefit from CSP, including those with non-left bundle branch block morphology, prior right ventricular pacing, or advanced structural heart disease. Comparative evaluation of HBP and LBBAP in dedicated trials will help clarify the optimal conduction system pacing modality across different clinical scenarios. Long-term data addressing lead performance, pacing thresholds, device longevity, and late complications are also required to inform clinical decision-making. As operator experience and technological advancements continue to evolve, conduction system pacing has the potential to transition from a niche bailout strategy to an integral component of personalised resynchronisation therapy in patients with heart failure.

## Conclusion

In this single tertiary centre experience, conduction system pacing proved highly feasible and effective as a rescue strategy in patients with unsuccessful conventional cardiac resynchronisation therapy. Despite a cohort characterised by advanced heart failure, severe left ventricular systolic dysfunction, and complex anatomical and electrical substrates, CSP was associated with a high procedural success rate and a favourable safety profile. Importantly, successful implantation resulted in significant electrical resynchronisation, reflected by marked QRS narrowing, and substantial improvement in left ventricular systolic function. These findings support the physiological premise that recruitment of the native His–Purkinje system can overcome many of the inherent limitations of coronary sinus-based epicardial pacing. In patients in whom conventional CRT cannot be delivered or has failed, CSP represents a clinically meaningful alternative with the potential to restore ventricular synchrony and improve cardiac performance.

## Data Availability

The original contributions presented in the study are included in the article/supplementary material. Further inquiries can be directed to the corresponding author.
