# Supplementary material for: Conduction system pacing as a rescue strategy for unsuccessful cardiac resynchronisation therapy implantation
Source: Front Physiol. 2026 Sep 16;17:1929170. doi: 10.3389/fphys.2026.1929170 (PMC13623700; doi:10.3389/fphys.2026.1929170)
Supplement: Supplementary file 1 [file DataSheet1.pdf]

CSP as a Rescue Strategy After Unsuccessful CRT

1. Clinical Problem: Unsuccessful Conventional CRT

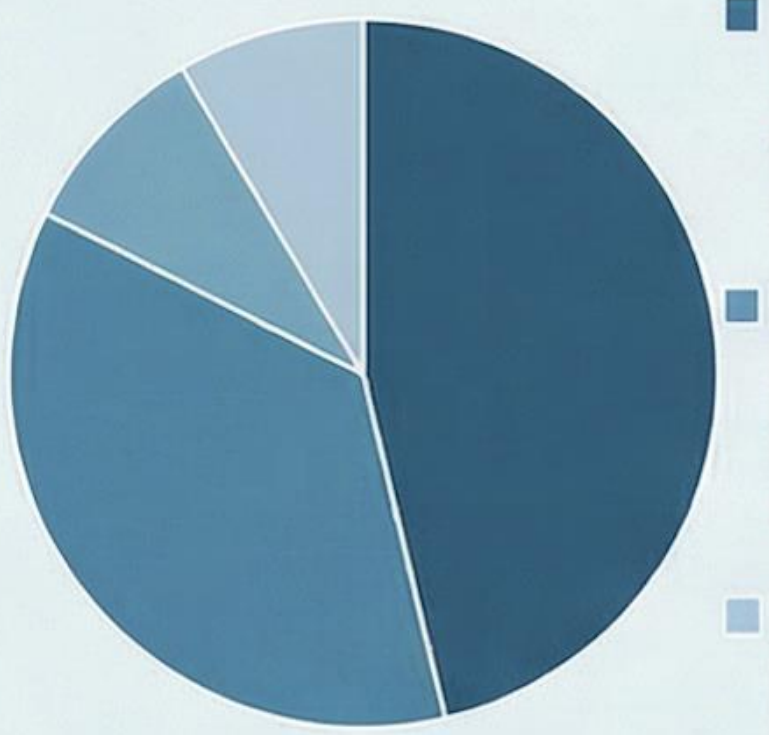

- **Failed Coronary Sinus (CS) Lead Implantation:** Primarily due to challenging anotorny of cannulation failure (47%)
- **Suboptimal LV Lead Performance:** Unsatisfactory parameters such as high capture thresholds or phrenic nerve stimulation (47%)
- **Procedural Barriers:** CIED infection (9%), CS dissection (3%), lead displacement (3%), and left-sided SVC (3%)

2. Study Design: Conduction System Pacing (CSP) as a Rescue Strategy

Single-Centre Retrospective Evaluation (N=32)

- Patients with heart fallure and reduced ejection fraction who failed conventional CRT attempts

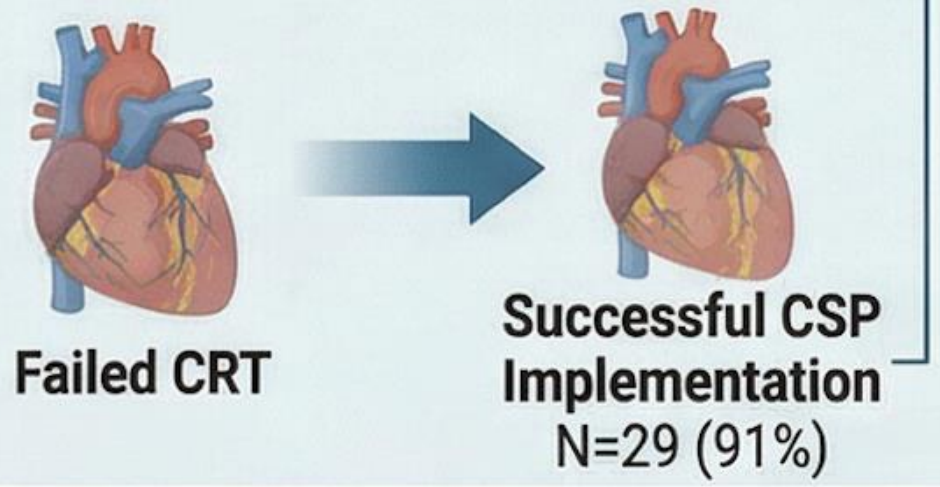

Successful CSP Implementation N=29 (91%)

- LBBAP (Left Bundle Branch Area Pacing): 72%
- HBP (His Bundle Pacing): 28%

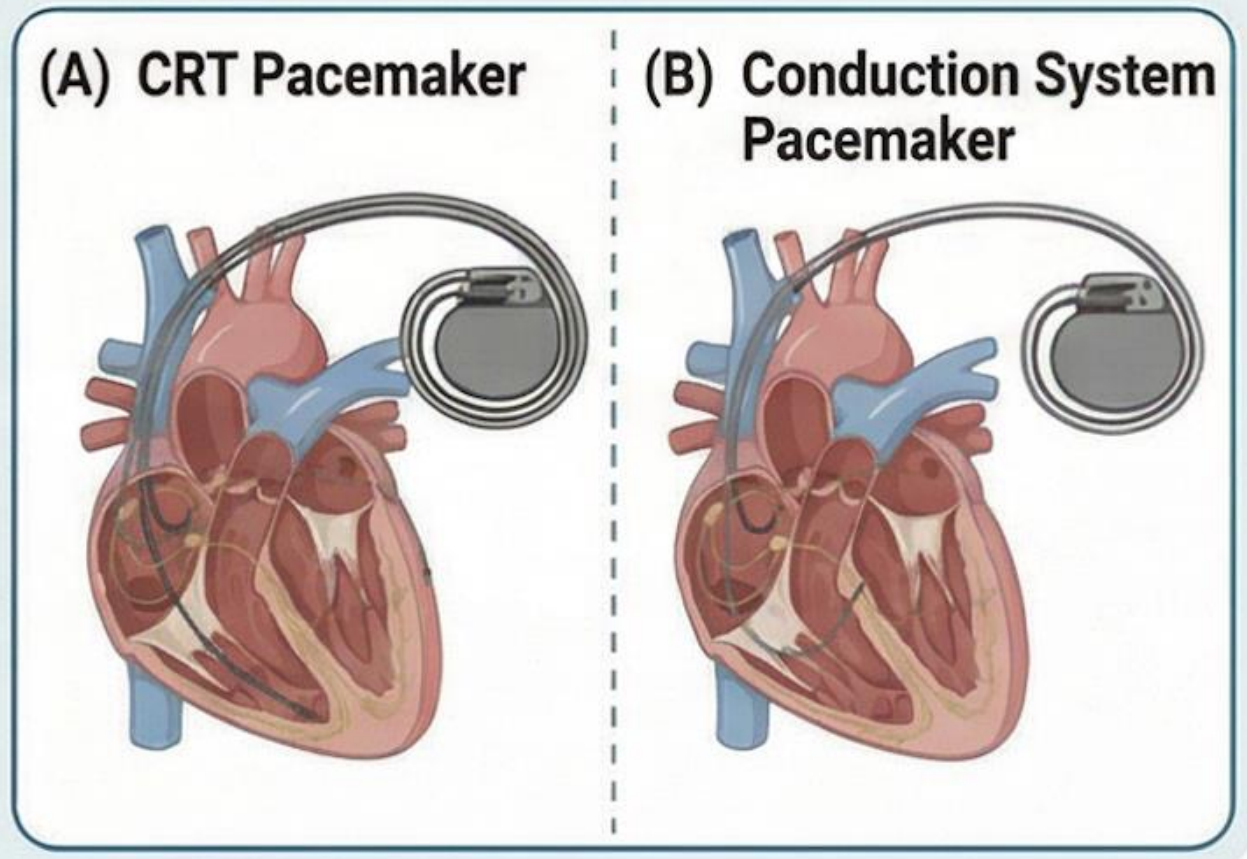

3. Key Results: Significant Resynchronisation & Remodelling

Electrical and Functional Improvement

Parameter	Baseline	Post-CSP	*p*-value
QRS Duration	165±38 ms	122±19.8 ms	p<0.0001
LVEF (%)	27±8.6%	47.7±11.2%	p<0.0001

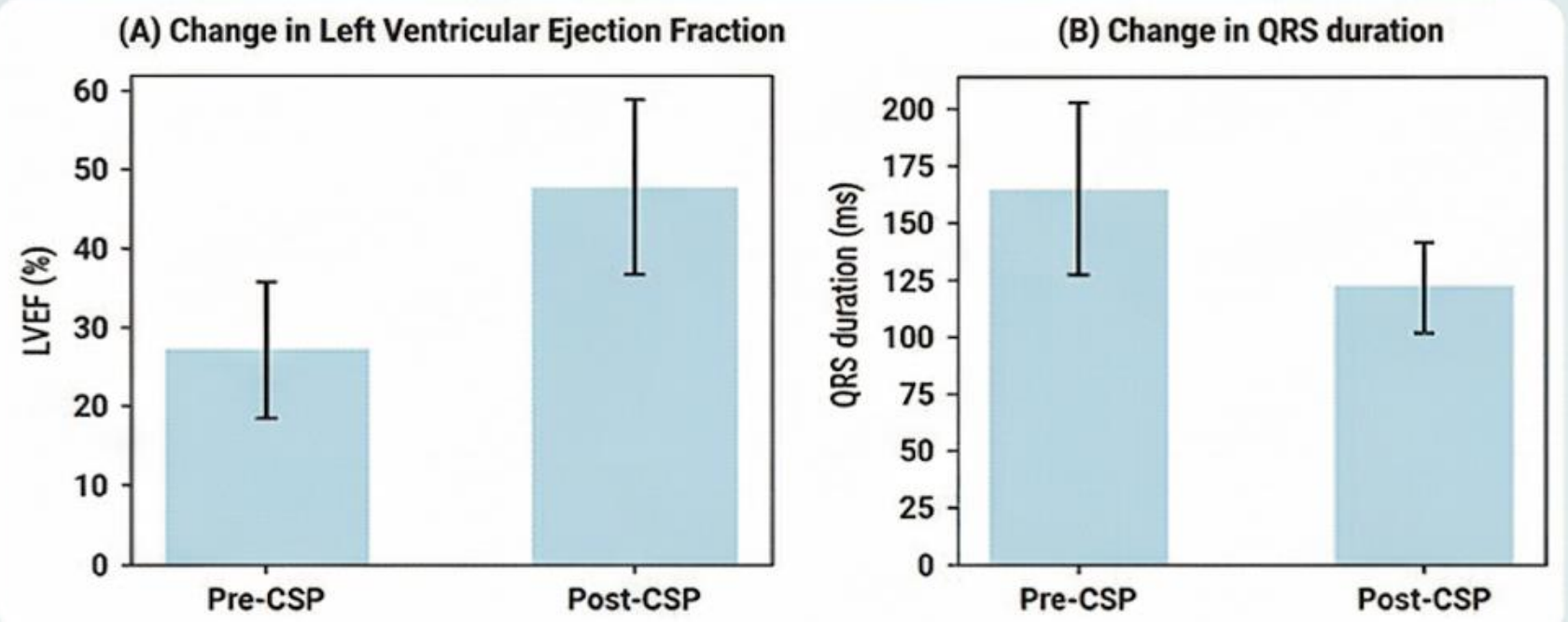

Safety and Stability

- **Lead Parameters:** Stable sensing and low capture thresholds (0.88±0.39 V at implant to 0.78±0.33 V at follow-up)
- **Complications:** Rare, only 1 procedure-related complication (pneumothorax) accurred

4. Clinical Outcomes

- **Mean Follow-up:** 641±522 Days
- **Mortality:** 3 patients (2 heart failure-related, 1 non-cardiac)
- **Hospitalisation:** 28% total follow-up admissions, with only 7% specific to heart failure
- **Device Integrity:** No evidence of device-related complications or late lead dysfunction in casse of clinical deterioration

5. Key Message

CSP is a feasible, safe, and highly effective rescue strategy for patients failing conventional CRT. It overcomes coronary sinus anatomical constraints to provide significant electrical resynchronisation and improvement in left ventricular systolic function.
